# Photobiomodulation as a regulator of neuroinflammation: mechanistic insights and therapeutic potential in brain disorders

**DOI:** 10.3389/fneur.2026.1902491

**Published:** 2026-10-16

**Authors:** Guillaume Blivet, Cédric Bertrand, David Brauge, Julien Delrieu, Jacques Touchon

**Affiliations:** 1 REGEnLIFE, Paris, France; 2 Clinique Universitaire du Sport, Toulouse University Hospital, Toulouse, France; 3 Service de Neurochirurgie, Clinique des Cèdres, Ramsay Santé, Cornebarrieu, France; 4 Maintain Aging Research Team, CERPOP, Université de Toulouse, Inserm, Université Paul Sabatier, Toulouse, France; 5 Gérontopôle, Department of Geriatrics, Toulouse University Hospital, Toulouse, France; 6 University of Montpellier, Montpellier, France; 7 JT Conseils, Montpellier, France

**Keywords:** brain-gut axis, clinical trials, low-level laser therapy, microglia, mitochondria, neurotechnology, oxidative stress, transcranial light therapy

## Abstract

Brain disorders, including neurodegenerative, neuropsychiatric, and cerebrovascular diseases, represent a growing global health challenge contributing to disability and mortality worldwide. Although these conditions differ in etiology and clinical presentation, accumulating evidence identifies neuroinflammation as a shared pathological mechanism. Involving microglial activation, astrocytic reactivity, cytokine dysregulation, oxidative stress, mitochondrial dysfunction, and blood–brain barrier disruption, it contributes to synaptic dysfunction, neuronal loss, and disease progression, yet therapeutic strategies targeting these processes remain limited. Photobiomodulation (PBM), the therapeutic application of low-intensity red to near-infrared light, has emerged as a neurotechnology capable of modulating biological pathways relevant to neuroinflammation. Preclinical studies suggest that PBM may influence mitochondrial activity through cytochrome c oxidase-mediated mechanisms, enhancing cellular metabolism while regulating reactive oxygen species, inflammatory signaling pathways, and tissue homeostasis. Through these effects, PBM may attenuate neuroinflammatory responses, regulate glial activation, and support neuroprotective processes across multiple neurological models. This narrative review examines evidence supporting neuroinflammation as a common denominator across major brain disorders and explores the biological rationale for PBM as a therapeutic strategy. It summarizes the cellular and molecular basis of PBM, its effects on neuroinflammatory pathways, and preclinical and clinical findings across brain disease models. Preclinical findings consistently support a multi-target anti-inflammatory effect, whereas clinical investigation remains at an early stage, with encouraging results in Alzheimer’s disease and multiple sclerosis but mainly negative data on primary outcomes in Parkinson’s disease and major depressive disorder. Further research is needed to optimize treatment parameters, clarify mechanisms of action, and determine clinical efficacy across disease contexts.

## 1 Introduction

Brain disorders are the leading cause of disability and the second leading cause of death worldwide, accounting for over 400 million disability-adjusted life years and 11 million deaths annually (1, 2). This burden continues to rise, driven by population aging for Alzheimer’s disease (AD) and Parkinson’s disease (PD) (3, 4), while stroke, traumatic brain injury (TBI) and major depressive disorders (MDD) remain major contributors across all age groups (2, 5, 6).

Despite advances in diagnosis and symptomatic care, most current treatments do not address the underlying disease mechanisms (5, 7, 8). Even emerging therapies targeting disease mechanisms often provide modest benefits or remain limited by accessibility and narrow therapeutic windows. Addressing this requires targeting the underlying disease mechanisms.

In recent years, neuroinflammation has emerged as a common pathological mechanism shared across these different brain disorders (9–11). Although initially protective, neuroinflammatory responses can become chronic and maladaptive, amplifying pathology across the broad spectrum of neurological conditions, positioning neuroinflammation as a promising therapeutic target for strategies aimed at modulating diseases mechanisms rather than isolated downstream symptoms (12, 13).

Photobiomodulation (PBM), the therapeutic application of low-intensity red to near-infrared light (typically 600–1,100 nm), has emerged as neurotechnology and a promising non-invasive approach capable of modulating neuroinflammatory responses (14, 15). Growing evidence suggests PBM may modulate, beside inflammatory responses, oxidative metabolism, cerebral blood flow, and neuroprotective processes by acting through mitochondrial and cellular signaling. Although the mechanistic basis remains to be fully elucidated, its favorable safety profile and broad biological relevance support PBM as an emerging therapeutic modality.

In this review, we examine neuroinflammation as a shared pathological mechanism across major brain disorders, and assess the biological rationale and current evidence supporting PBM as a candidate therapeutic strategy.

## 2 Neuroinflammation as a central driver of brain disorders

### 2.1 Neuroinflammatory mechanisms

Neuroinflammation is a protective CNS response to injury that, when dysregulated, becomes chronic and maladaptive, contributing to synaptic dysfunction and neuronal damage (10, 16). Microglial cells are the main drivers of this response, with astrocytes playing a key modulatory role (17–19). Reactive microglia release pro-inflammatory cytokines and reactive oxygen species that, when sustained, engage effectors such as NF-κB and the NLRP3 inflammasome in a self-reinforcing loop that perpetuate inflammation (20). Chronic neuroinflammation is closely intertwined with oxidative stress, mitochondrial dysfunction (21) and blood–brain barrier (BBB) disruption, which lead to peripheral immune cells infiltration in the CNS, further amplifying the inflammation (22).

Whatever the initiating factor, protein aggregates, mechanical or ischemic injury, autoimmune attack, or systemic and environmental stressors, these responses converge on a shared set of intracellular effectors in which redox imbalance, dysregulated nitric oxide (NO), and altered calcium handling amplify cytokine production and sustain glial reactivity (20, 21, 23, 24).

### 2.2 Neuroinflammation across diseases

Although neuroinflammation is shared across the disorders listed in Table 1, its triggers differ considerably depending on the disease context. In AD and PD, protein aggregates, respectively amyloid-β/tau and α-synuclein, act as damage-associated molecular patterns capable of triggering microglial reactivity (25–28). In MS, a peripheral autoimmune response breaches the BBB and targets myelin, recruiting microglia and astrocytes that amplify injuries (29–31). In MDD, persistent low-grade inflammation affects monoaminergic neurotransmission, neuroplasticity, and the hypothalamic–pituitary–adrenal axis (32, 33). In TBI and stroke, secondary inflammatory cascades amplify the initial mechanical or ischemic injury, disrupt the BBB, and impair recovery (32, 34–37).

**Table 1:** Neuroinflammation across major brain disorders: disease-specific triggers, shared inflammatory mechanisms, and reported effects of photobiomodulation.

Disease	Primary trigger	Inflammatory mechanism	Clinical/pathological consequence	Reported effects of photobiomodulation
Alzheimer’s disease (AD)	Amyloid-β plaques and hyperphosphorylated tau acting as DAMPs	Chronic microglial reactivity and astrocytic activation; release of pro-inflammatory cytokines (TNF-α, IL-1β, IL-6); NLRP3 inflammasome engagement; oxidative stress	Synaptic dysfunction, hippocampal and cortical neuronal loss, progressive cognitive decline	*Preclinical*: **↓** Aβ/tau, ↓ microglial reactivity and pro-inflammatory cytokines, ↓ NLRP3 with anti-inflammatory microglial polarization; restored mitochondrial function; attenuated BBB disruption. Brain-gut/gut-targeted PBM modulate microbiota and hippocampal neuroinflammation. *Clinical*: cognitive improvement (MMSE, ADAS-Cog) with ↑ BDNF; favorable safety and compliance; no direct inflammatory biomarker reported; rigorous trials ongoing (TRAP-AD, Light4Life).
Parkinson’s disease (PD)	α-synuclein aggregates acting as DAMPs	Sustained microglial reactivity in the substantia nigra; pro-inflammatory cytokine release; mitochondrial dysfunction and oxidative stress in dopaminergic neurons	Selective loss of nigrostriatal dopaminergic neurons; motor and non-motor symptoms	*Preclinical*: MPTP-insult neuroprotection, preserved nigral dopaminergic neurons and mitochondrial integrity; improved locomotion; peripheral delivery comparable to transcranial. *Clinical*: sham-controlled trials largely negative on primary motor outcomes.
Multiple sclerosis (MS)	Peripheral autoimmune response; autoreactive lymphocytes breaching the BBB and targeting myelin	Demyelination with secondary recruitment of microglia and astrocytes; sustained pro-inflammatory signaling, oxidative stress	Demyelinating lesions, progressive axonal loss, motor/sensory/cognitive decline	*Preclinical*: (EAE) ↓ clinical severity; spinal cytokine profile shifted toward a regulatory state; peripheral immune cell infiltration prevention. *Clinical*: ↑ serum IL-10 (nitrites unchanged); preliminary functional benefits.
Major depressive disorder (MDD)	Chronic stress; persistent low-grade peripheral inflammation	Elevated pro-inflammatory cytokines (IL-6, TNF-α) acting as molecular mediators; HPA axis dysregulation; impaired monoaminergic neurotransmission; reduced neuroplasticity	Depressive symptoms, anhedonia, cognitive impairment, treatment resistance	*Preclinical*: brain-gut PBM restores cognition, hippocampal Sirt1 and CA1 dendritic spine density, and modulates microglial morphology and gut microbiota. *Clinical*: discordant findings; moderate symptom reduction in meta-analysis versus negative primary outcome (ELATED-3); benefit limited to sleep quality.
Traumatic brain injury (TBI)	Mechanical primary injury	Secondary inflammatory cascades; microglial reactivity with phenotypic imbalance; cytokine and ROS release; BBB disruption; peripheral immune cell infiltration	Lesion expansion, neuronal loss, long-term cognitive, motor, and behavioral deficits	*Preclinical*: ↓ secondary neuroinflammation through glial and oxidative modulation; inhibited microglial activation and improved cognitive and motor recovery. *Clinical*: cognitive improvement in small studies; adequately powered sham-controlled trials scarce.
Stroke	Ischemic or hemorrhagic insult	Acute microglial and astrocytic reactivity; pro-inflammatory cytokine release (TNF-α, IL-1β, IL-6); systemic inflammatory response; BBB breakdown	Infarct expansion, impaired functional recovery, post-stroke cognitive impairment	*Preclinical*: inhibition of neurotoxic astrocytic polarization and neuronal protection. *Clinical*: early phase II signals not confirmed. Pivotal phase III trial halted for futility (NEST-3, *n* = 630), with no difference from sham on 90-day functional outcome.

Aβ, amyloid-β; ADAS-Cog, Alzheimer’s Disease Assessment Scale–Cognitive subscale; BBB, blood–brain barrier; BDNF, brain-derived neurotrophic factor; DAMP, damage-associated molecular pattern; EAE, experimental autoimmune encephalomyelitis; HPA, hypothalamic–pituitary–adrenal; IL, interleukin; MMSE, Mini-Mental State Examination; MPTP, 1-methyl-4-phenyl-1,2,3,6-tetrahydropyridine; NLRP3, NLR family pyrin domain containing 3; PBM, photobiomodulation; ROS, reactive oxygen species; Sirt1, sirtuin 1; TNF-α, tumor necrosis factor alpha.

Beyond the CNS, systemic inflammation also modulates the neuroinflammatory response by compromising the integrity of the BBB, facilitating the entry of peripheral immune cells and mediators into the brain, and sustaining glial reactivity across conditions including stroke, MS, and AD (38–40). A key component of this crosstalk between the periphery and the CNS is the brain-gut-microbiota axis: growing evidence highlights the role of gut dysbiosis in the pathophysiology of AD, PD, MS, stroke, and MDD, modulating neuroinflammation through increased intestinal permeability, microbial metabolites, and vagal afferent pathways (41–43). Preclinical models further suggest the gut microbiota influence microglial maturation and their inflammatory phenotype (44). Together, these observations indicate that neuroinflammation extends beyond the CNS to the periphery and the gut microbiota, implying that peripheral or gut-associated interventions may influence central inflammation without direct access to the brain tissue.

## 3 Biological basis of photobiomodulation

PBM, formerly known as low-level laser therapy, refers to the therapeutic application of non-ionizing, non-thermal red to near-infrared light (typically 600–1,100 nm) to biological tissues (45, 46). When applied to the brain, PBM is usually delivered transcranially (tPBM), but photon penetration is attenuated by overlying layers (hair, scalp, blood, skull bone) before reaching brain tissue (47). PBM also follows a biphasic dose–response, in which low and high levels of light exert opposing effects (48, 49). As a result, wavelength selection, dosimetry, and anatomical targeting are critical determinants of therapeutic efficacy (50).

To overcome these limitations, peripheral delivery strategies have emerged. Gordon et al. (51) compared peripheral PBM targeting the abdomen or leg with tPBM in mouse and non-human primate PD models, finding comparable neuroprotection. This may reflect an abscopal-like effect, whereby irradiated tissues release circulating mediators that modulate immune and metabolic responses at distant sites. Other studies have focused more specifically on the gut-brain axis by combining tPBM with abdominal PBM. In a mouse model of AD, the combined brain-gut PBM treatment produced a neuroprotective effect characterized by memory restoration and the normalization of key AD markers (Aβ1–42, pTau), oxidative stress, and neuroinflammation greater than in the tPBM group (52). Similar effects were observed in a chronic unpredictable mild stress mouse model of MDD, where brain-gut PBM restored cognitive function and hippocampal Sirt1 levels, attenuated neuroinflammation, and partially corrected stress-induced gut dysbiosis (53). Together, these observations support the view that neuroinflammation can be therapeutically targeted outside the brain, and provide a preclinical rationale for combined brain-gut PBM strategies in brain disorders.

Although PBM mechanisms remain to be fully understood, converging preclinical evidence supports a model centered on mitochondrial photoreception (Figure 1), in which photons are absorbed by cytochrome c oxidase (CCO), enhancing mitochondrial activity and adenosine triphosphate (ATP) production while modulating intracellular calcium (Ca^2+^), reactive oxygen species (ROS), NO availability, and cyclic adenosine monophosphate (cAMP)-related signaling (49, 54). Transient ROS modulation, together with activation of pathways such as ERK, PI3K/Akt, and Sirt1, influences transcription factors, including NF-κB and Nrf2 that are central to inflammatory regulation (49, 55). A growing body of evidence further suggests that CCO is not the sole photoacceptor involved in the mechanisms underlying PBM. Other non-mitochondrial chromophores, including heat- and light-gated transient receptor potential (TRP) ion channels may contribute at specific PBM wavelengths (56). These non-mitochondrial mechanisms, including the proposed role of structured intracellular water, remain hypothetical and await direct experimental validation. Clarifying these pathways is essential to refine PBM mechanisms and inform wavelength- and dose-specific strategies.

**Figure 1:**
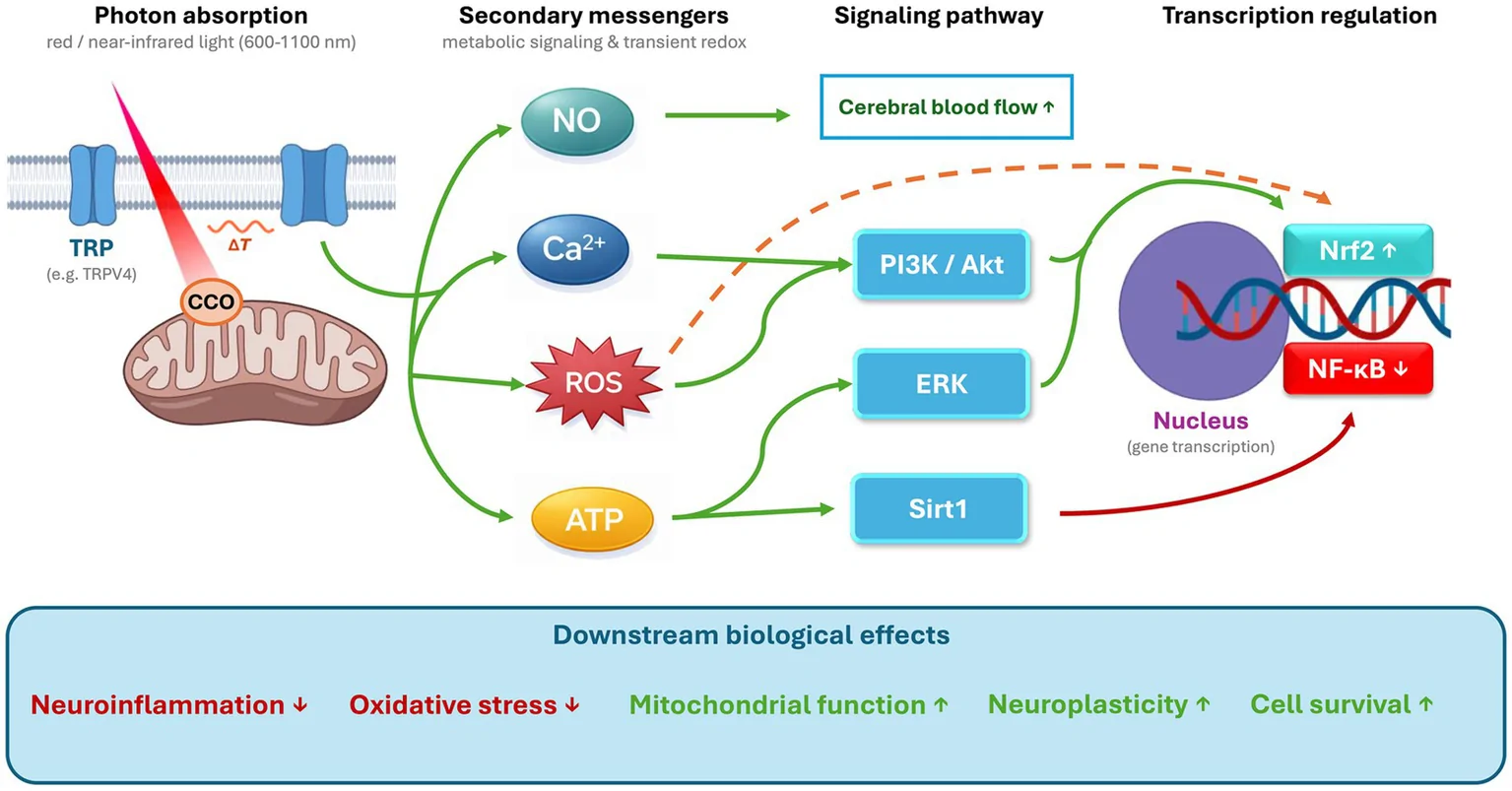
From photon absorption to neuroinflammatory modulation: Intracellular signaling cascade of photobiomodulation. Red to near-infrared light (600–1,100 nm) is absorbed primarily by cytochrome c oxidase (CCO), complex IV of the mitochondrial electron transport chain, and may additionally engage non-mitochondrial photoacceptors such as transient receptor potential (TRP) channels (e.g., TRPV4). Photon absorption modulates secondary messengers: nitric oxide (NO), intracellular calcium (Ca^2+^), reactive oxygen species (ROS), and adenosine triphosphate (ATP); photodissociation of NO from CCO relieves its inhibition and promotes mitochondrial respiration and cerebral blood flow. Other messengers activate downstream pathways (PI3K/Akt, ERK, Sirt1) that regulate key transcription factors, activating Nrf2 (antioxidant defenses) and inhibiting NF-κB (pro-inflammatory signaling). Through these converging pathways, PBM is proposed to reduce neuroinflammation and oxidative stress while enhancing mitochondrial function, cerebral blood flow, neuroplasticity, and cell survival. Some images were created with Biorender.

## 4 PBM effects on neuroinflammation

### 4.1 Modulation of microglial and astrocyte reactivity

Microglial polarization is among the most consistently reported targets of PBM. *In vitro* and *in vivo* studies have shown that PBM is capable of shifting the phenotype of reactive microglia from a pro-inflammatory profile, characterized by, but not limited to, elevated levels of iNOS, TNF-α, and IL-1β, to a more regulatory state expressing Arg-1 and IL-10 (57, 58). Similar modulatory effects have been reported in astrocytes, where PBM inhibits the neurotoxic polarization of these cells, preserves synaptic integrity, and promotes neuronal survival (57, 59).

### 4.2 Inhibition of NLRP3 inflammasome-driven cytokine amplification

The Nod-like receptor protein 3 (NLRP3) inflammasome is able to activate caspase-1 to produce inflammatory cytokines, such as IL-1β and IL-18, and therefore has been considered a key contributor to neuroinflammation (60). In an APP/PS1 mouse model of AD, PBM reduced NLRP3 protein in both microglia and astrocytes and was associated with regulated secretion of inflammatory factors, microglial anti-inflammatory phenotypic polarization and enhanced Aβ clearance (61). In reactive microglia and macrophages, PBM has also been reported to attenuate NF-κB signaling (62, 63) although the effect of PBM on NF-κB appears to be contact-dependent, with both inhibitory and stimulatory responses described depending on baseline cellular activation state (49). In parallel, transient PBM-induced ROS modulation activates Nrf2 pathway, upregulating endogenous antioxidant responses (64, 65). Together, the modulation of NLRP3-driven cytokine amplification and reinforcement of Nrf2-dependent antioxidant defenses positions PBM as a modulator of multiple nodes of chronic neuroinflammation.

### 4.3 Cerebrovascular protection and neurotrophic support

Beyond these direct effects on glial cells and their signaling pathways, PBM also acts on mechanisms that contribute to the structural and functional protection of neurons in an inflammatory context. BBB disruption, an early and shared feature of multiple neuroinflammatory conditions, has been shown to be attenuated by PBM through upregulation of tight junction proteins (66), and restoration of barrier function via modulation of endothelial vWF in a humanized hypoxic BBB model (67). PBM has also been associated with upregulation of brain-derived neurotrophic factor (BDNF), supporting neuronal survival and synaptic plasticity (68). Together, these observations indicate that by coupling reduced inflammatory signaling with enhanced neurotrophic support, PBM may address both the injurious and reparative dimensions of neuroinflammatory pathology.

## 5 Evidence from preclinical and clinical studies

### 5.1 Preclinical evidence

The preclinical literature on PBM in neurological disease models has expanded substantially over the past decade, providing a mechanistic foundation for its therapeutic potential across multiple conditions. In AD models, tPBM and peripheral PBM have been associated with reduced Aβ plaque and tau hyperphosphorylation. A reduction in microglial reactivity and an improvement in cognitive performance have also been demonstrated, accompanied by restored mitochondrial function, reduced oxidative stress, and decreased pro-inflammatory cytokines release (61, 66, 69, 70). Convergent preclinical evidence indicates that combined brain-gut or gut-targeted PBM normalizes behavioral deficits, attenuates hippocampal neuroinflammation, and reduces amyloid/tau burden in AD mouse models, with concomitant modulation of gut microbiota composition (52, 71, 72). In PD models, peripheral PBM has been shown to confer neuroprotection against MPTP-induced injury, preserving dopaminergic neurons in the substantia nigra, improving locomotor function, and protecting mitochondrial integrity with outcomes comparable to those achieved by transcranial delivery (51, 73). In MS, PBM has demonstrated anti-inflammatory and neuroprotective effects in EAE model of the pathology, including reduced clinical disease severity, modulation of spinal cord cytokine profiles toward a regulating state, and prevention of peripheral immune cell infiltration into the spinal cord (74–76). In TBI and stroke models, reduced secondary neuroinflammation through glial reactivity and oxidative stress modulation was associated with improvements in cognitive and motor recovery after tPBM treatment (77, 78). In MDD-related models, combined brain-gut PBM rescued cognitive impairments in chronically stressed mice, alongside restoration of hippocampal Sirt1 levels, preservation of dendritic spine density in the CA1, modulation of microglial morphology, and modulation of gut microbiota composition (53). Across these conditions, the preclinical literature reveals a consistent anti-inflammatory and neuroprotective signature, suggesting that PBM engages conserved cellular mechanisms rather than disease-specific pathways.

A key limitation is the heterogeneity of PBM protocols and incomplete dosimetric reporting, which makes dose–response characterization unreliable (49, 50).

### 5.2 Clinical evidence

Clinical investigation of PBM remains at an early stage but has rapidly expanded over the past decade. Most trials are limited by small samples and methodological heterogeneity, so current evidence should be interpreted cautiously (79).

In AD and mild cognitive impairment (MCI), cognitive benefits have been reported following PBM treatment. In 2017, five patients with mild-to-moderate dementia improved on MMSE and ADAS-Cog after 12 weeks of combined transcranial and intranasal PBM, but declined sharply during the 4-week follow-up period (80). A more recent investigation in MCI and mild AD patients documented cognitive improvements alongside increased serum BDNF after 12 weeks of active tPBM treatment (81). A randomized, double-blind, sham-controlled pilot trial of brain-gut PBM enrolled 53 patients with mild-to-moderate AD and demonstrated safety, high treatment compliance, and trends toward cognitive improvement (82). To further assess the efficacy of PBM therapy in AD patients, two ongoing randomized clinical trials are evaluating multiple outcomes. The TRAP-AD trial (NCT04784416) is recruiting 196 patients and is focusing on the dose-dependent effects of PBM in early AD and amnestic MCI. In parallel, the multicentric pivotal Light4Life trial (NCT05926011) is currently recruiting 108 patients, evaluating a six-month brain-gut PBM intervention with clinical and biological outcomes including cognitive parameters, autonomy and inflammatory and neurodegenerative biomarkers.

In PD, recent randomized sham-controlled trials have been largely negative on primary motor outcomes. A double-blind, randomized, sham-controlled pilot study in 22 patients found no significant difference between active and sham treatment (83). Similarly, a double-blind, randomized, sham-controlled feasibility trial treating 20 participants with tPBM for 12 weeks showed no significant difference between groups at any assessment point (84). The largest sham-controlled study to date, with 63 participants and an 8-week intervention, did not detect significant differences between active and sham groups on the primary motor outcome except in stage 3 where participants had the choice of continuing at-home tPBM (85). These findings emphasize the importance of dosimetric optimization and adequately powered designs before efficacy can be established.

In MS, a randomized clinical trial in relapsing–remitting MS patients reported increased serum IL-10 but no effect on nitrite levels (86), while a systematic review identified preliminary benefits of PBM on motor, sensory, and cognitive function (87).

Regarding TBI, a systematic review of small clinical studies has reported cognitive improvements following tPBM in chronic TBI patients (88), but adequately powered, multicentric sham-controlled trials remain scarce.

In stroke, the NEST program provides the largest clinical dataset in the field. An initially positive phase II trial (NEST-1) was followed by a phase III trial that did not confirm efficacy (NEST-2) (89, 90), and the pivotal NEST-3 trial (*n* = 630) was subsequently halted for futility, showing no difference from sham on 90-day functional outcome (adjusted OR 1.024; 95% CI 0.705–1.488) (91). This failure has been attributed in part to insufficient light delivery, a single treatment session, and the absence of dosimetric verification at the target tissue. These limitations reinforce the methodological concerns discussed below.

In MDD, observations may be the most discordant compared with other brain disorders. A recent meta-analysis of randomized trials reported moderate effect size in reduction of depressive symptoms following tPBM compared with sham (92). However, the ELATED-3 multicentric sham-controlled trial did not observe any differences between tPBM and sham for its primary outcomes (HDRS-17) among the 49 MDD subjects, and the authors concluded that a minimum dosimetric threshold is likely required for clinical benefit (93). Most recently, a tPBM sham-controlled trial demonstrated significant improvement in sleep quality (PSQI) but no significant difference on depression severity scales (94).

Across all conditions, PBM has shown a consistently favorable safety profile, with no serious adverse events reported. Nevertheless, long-term safety data remain limited, highlighting the need for extended follow-up studies. Beyond radiometry standardization, the systematic integration of mechanistic biomarkers, including blood-based biomarkers of neuroinflammation and mitochondrial function and neuroimaging of cerebral metabolism, will be essential to link PBM’s mechanistic plausibility to reproducible clinical benefit and identify the patient populations most likely to respond.

## 6 Key challenges and future directions

Despite this supportive evidence, several challenges must be addressed in order to demonstrate the relevance of PBM in the treatment of brain disorders.

The foremost issue is dosimetric heterogeneity across studies (49, 50). Parameters such as wavelength, irradiance measurement, pulse frequency, and session duration and frequency vary considerably, making comparisons and dose–response characterization unreliable. This challenge is compounded by the incomplete reporting of stimulation protocol (50). Despite this heterogeneity, most studies reporting neuroprotective effects have used wavelengths in the 660–850 nm range, irradiances of approximately 10–100 mW/cm^2^, and repeated sessions over several weeks. Systematically disclosing these parameters would improve cross-study comparison and reproducibility.

PBM’s mechanisms also remain incompletely defined. Although CCO activation is the most widely accepted primary mechanism (49, 54), other pathways, including direct photomodulation of ion channels, membrane photoreceptors, and non-mitochondrial chromophores (56), are poorly characterized. Clarifying them is necessary to optimize treatment parameters and to refine our understanding of where PBM intervenes within the neuroinflammatory cascade.

A further obstacle to clinical translation is the absence of validated biomarkers to confirm target engagement and quantify PBM’s biological effects. Most trials rely on cognitive and behavioral outcomes (79–85), which offer little mechanistic insight concerning *in vivo* neuroinflammation modulation. Validated biomarkers would support efficacy assessment and link biological responses to delivered light parameters. Current options are limited: CSF markers (cytokines, chemokines, oligoclonal bands, IgG index) are invasive, and microglial PET tracers (TSPO ligands such as 11C-PBR28, 18F-DPA-714, or 18F-GE-180) are expensive. Emerging blood-based biomarkers such as GFAP may facilitate mechanistic assessment of PBM in future trials (95). Neuroglobin represents another candidate of mechanistic interest. This neuronal oxygen-binding globin preserves mitochondrial function and limits free radical accumulation (96, 97), and its elevation attenuates neuroinflammatory changes in experimental models (98). Its detection in umbilical cord blood, where concentrations correlate with hemoglobin, further indicates that neuroglobin is quantifiable in peripheral blood and physiologically regulated by oxygen availability (99).

A final methodological limitation concerns light delivery itself: the limited penetration of tPBM into the brain warrants further investigation. In conditions with diffuse neuroinflammation, tPBM may prove insufficient to achieve the desired therapeutic effects, and peripheral PBM, particularly transabdominal PBM acting through the gut-brain axis (51, 52, 72), offers a coherent mechanistic alternative to be investigated in clinical trials.

Beyond these methodological considerations, two strategic directions will shape the clinical development of PBM. The first is identifying where it fits within each disease: establishing at which stage and through which mechanism PBM acts would help determine the conditions and patient subgroups most likely to benefit. The second follows from this mechanistic positioning: because PBM acts on upstream pathways distinct from the symptomatic targets of current therapies, it may be best suited as an add-on rather than a standalone treatment. Finally, the possibility that PBM is ineffective for certain indications, notably motor symptoms in Parkinson’s disease, must be entertained rather than attributed solely to suboptimal dosimetry.

## 7 Conclusion

Neuroinflammation has emerged as a pathological denominator shared across diverse brain disorders, opening a rationale for trans-nosological therapeutic strategies. Current evidence indicates that PBM modulates key neuroinflammatory pathways across experimental models, while clinical translation remains at an early stage and will require adequately powered controlled trials to establish efficacy. Beyond its potential as a stand-alone intervention, PBM may also serve as an adjunctive and synergistic therapy alongside existing treatments, warranting further investigation in combination with conventional therapeutic strategies. A better understanding of the role of PBM within disease pathophysiology will be essential to identify optimal targets, patient populations, and treatment windows. Continued efforts in mechanistic research, biomarker development, dosimetry standardization, and large-scale clinical trials are needed to fully realize the therapeutic potential of PBM.
